# Supplementary figures and images for: Metabolic reprogramming tips vaccinia virus infection outcomes by stabilizing interferon-γ induced IRF1
Source: PLoS Pathog. 2024 Oct 30;20(10):e1012673. doi: 10.1371/journal.ppat.1012673 (PMC11554218; doi:10.1371/journal.ppat.1012673)

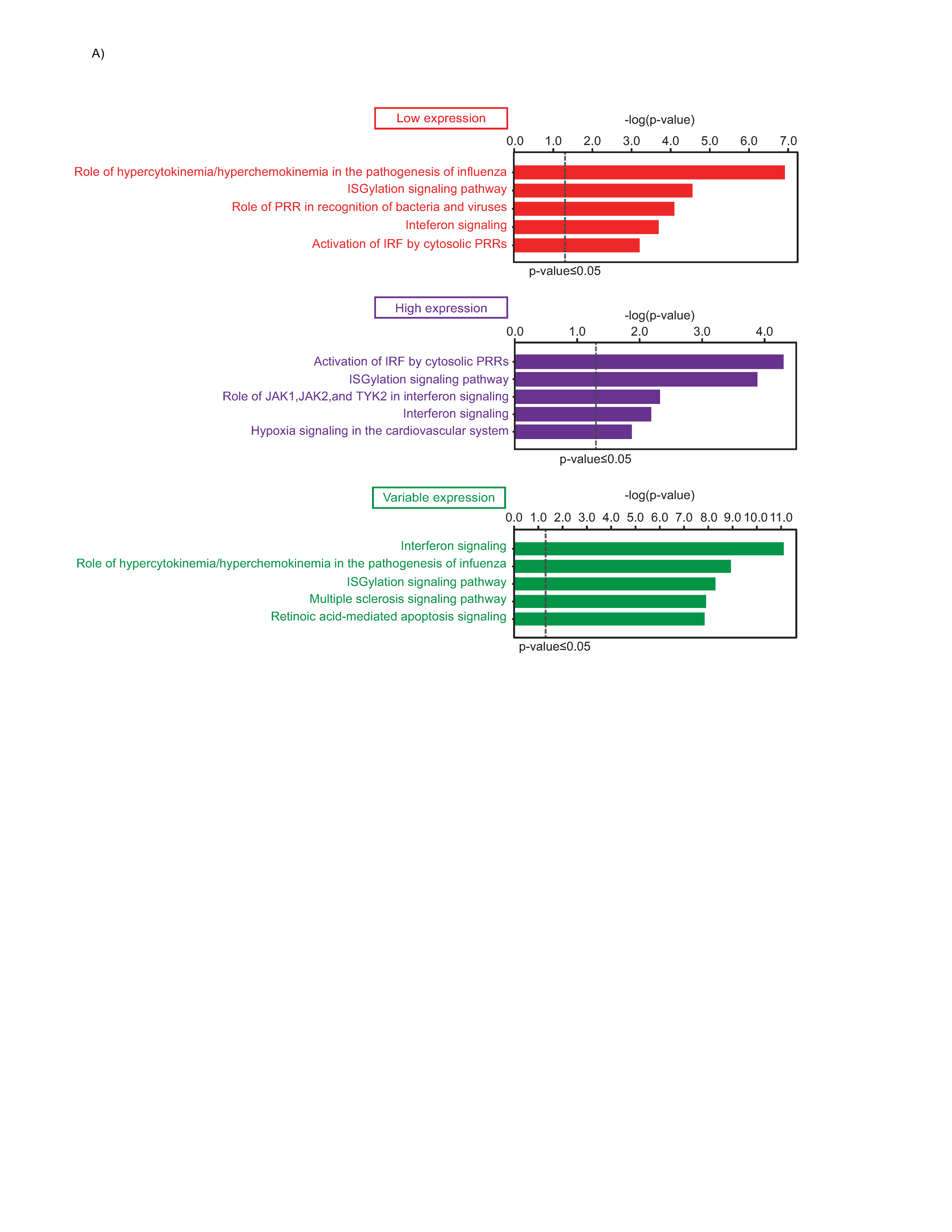

Supplement: S1 Fig — IPA core canonical pathway analysis was performed on forty-seven core ISGs clustered into three categories (low, high and variable) based on expression patterns across tissues (Fig 1). Dashed line indicates a p-value significance threshold of 0.05. (TIF) [file ppat.1012673.s001.tif]

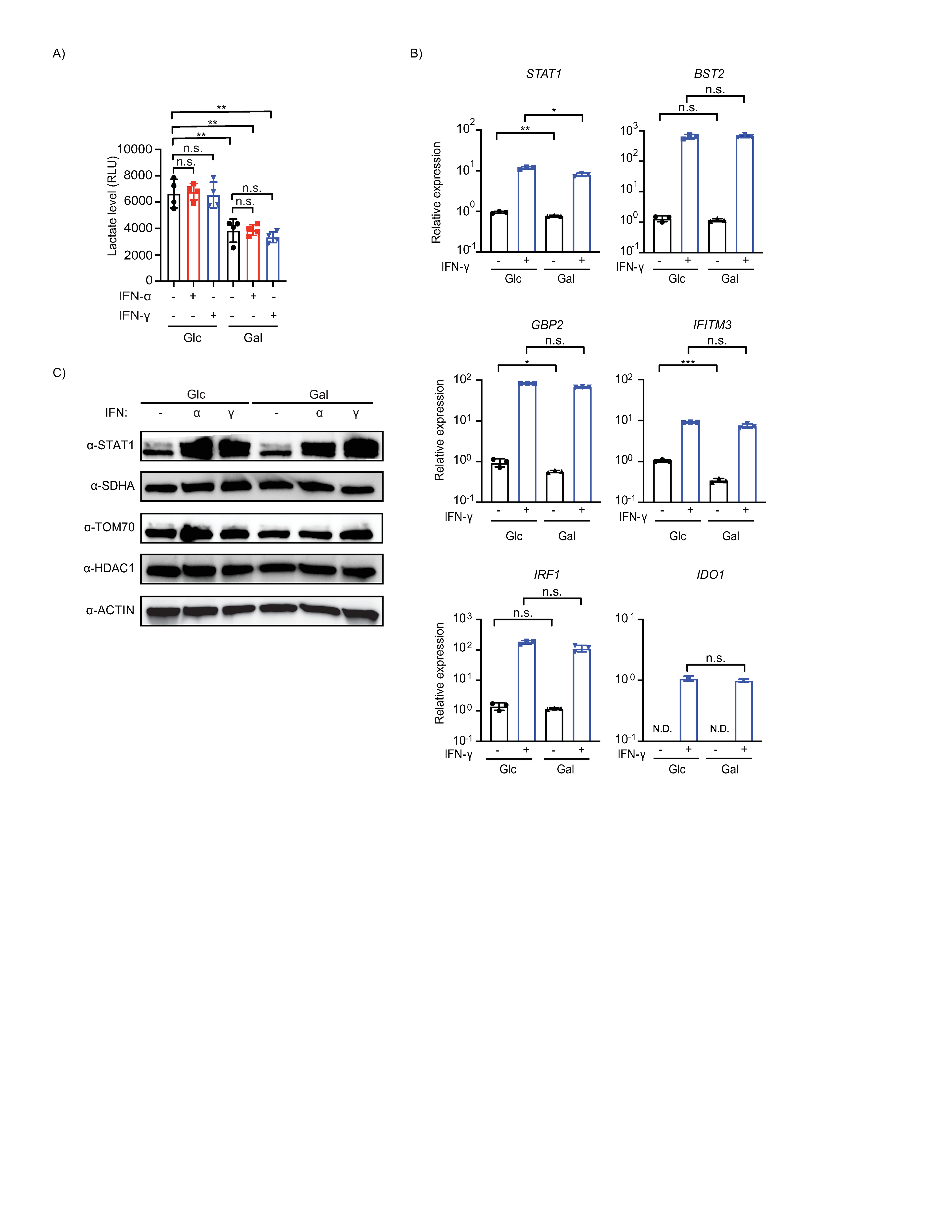

Supplement: S2 Fig — (A) Lactate levels for A549 cells grown in glucose/galactose with and without IFN-priming. Assay was performed using Lactate-Glo (N = 4). (B) qPCR of canonical ISGs relative to β-actin for cells pre-treated with IFN-γ, grown in glucose or galactose (N = 3). (C) Western blot analysis of non-ISG proteins from different cellular compartments in IFN-primed cells grown in glucose or galactose. Statistical analysis was performed using an unpaired t-test in GraphPad Prism 9.5.1: n.s. not significant, * P ≤ 0.05, ** P < 0.01, *** P ≤ 0.001, **** P ≤ 0.0001. Expression is ordered by the ratio of RNA expression for each gene shown in glucose compared to galactose. (TIF) [file ppat.1012673.s002.tif]

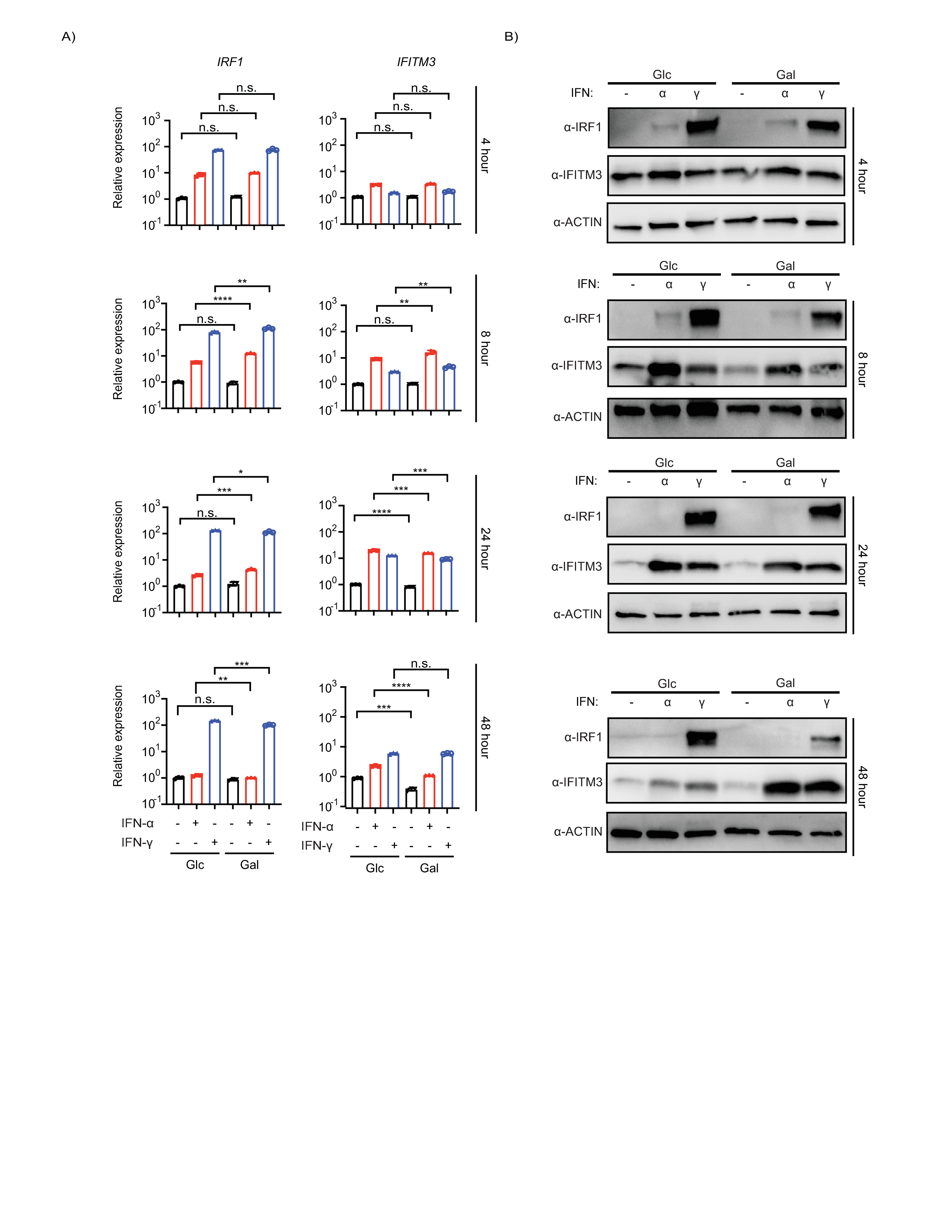

Supplement: S3 Fig — A549 cells in glucose or galactose supplemented media were treated with either interferon-α or interferon-γ. RNA and protein samples from the indicated time points (hours: 4,8, 24, 48) were harvested and analyzed for IRF1 and IFITM3 RNA and protein expression. (A) Relative abundance of IRF1 and IFITM3 transcripts by qPCR across indicated time points post-IFN treatment in Glc/Gal groups (N = 3). (B) Western blot analysis of IRF1 and IFITM3 protein expression at indicated times post-IFN treatment in Glc/Gal. Statistical analysis was performed using an unpaired t-test in GraphPad Prism 9.5.1: n.s. not significant, * P ≤ 0.05, ** P < 0.01, *** P ≤ 0.001, **** P ≤ 0.0001. (TIF) [file ppat.1012673.s003.tif]

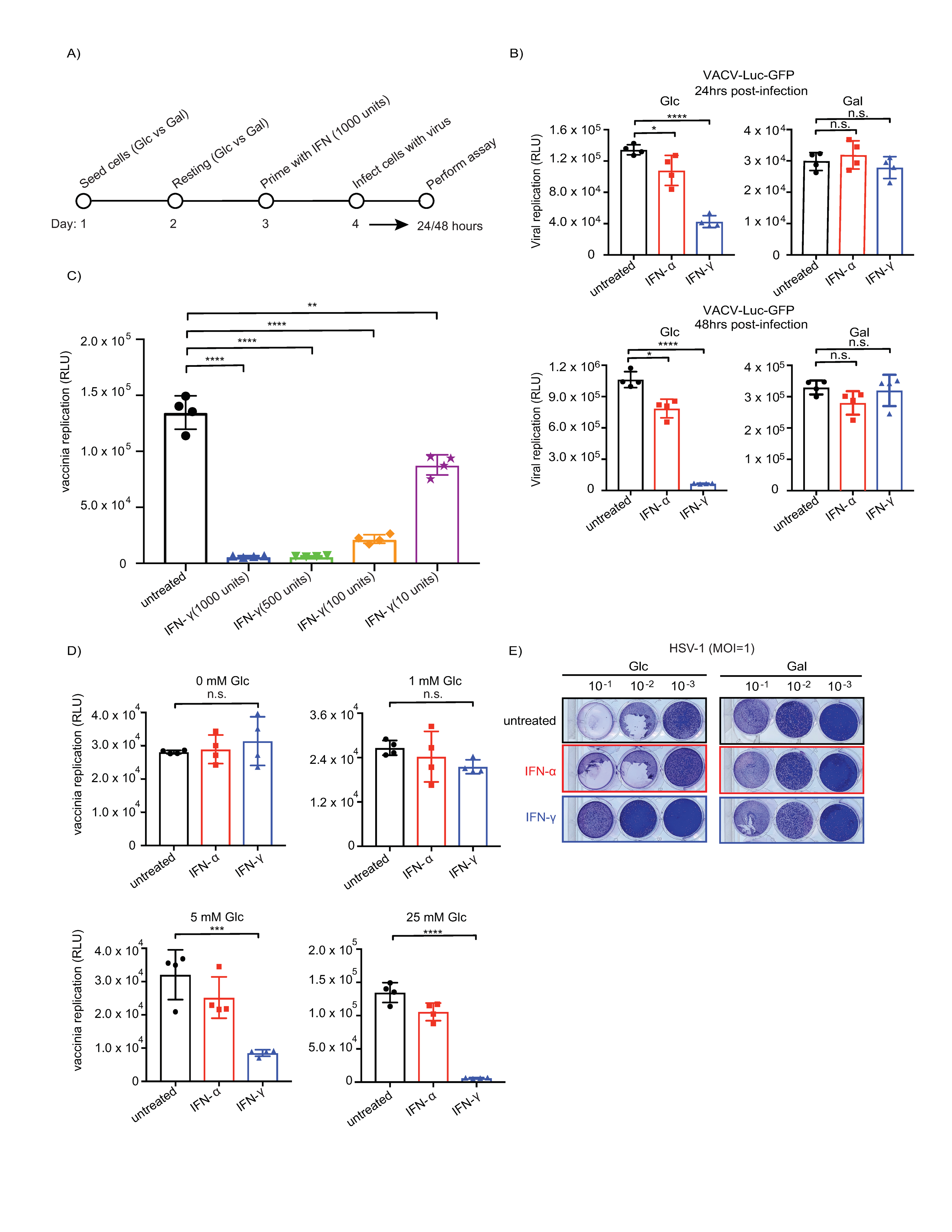

Supplement: S4 Fig — (A) Diagrammatic view of experimental set-up in A549 cells and timeline. (B) Vaccinia virus replication (luciferase) assays. Luciferase assays of A549 cells infected with VACV-Luc-GFP grown in either glucose (25 mM) or galactose (10 mM); 24 hours (top) and 48 hours post-infection (bottom). (C) Vaccinia virus replication (luciferase) assays over IFN-γ concentrations. Cells were primed for 24 hours with 1000, 500, 100, 10, or 0 units of IFN-γ followed by infection with VACV-Luc-GFP. (D) Vaccinia virus replication (luciferase) assays over glucose concentrations. Cells were grown in 0, 1, 5, 25 mM of glucose and primed with 1000 units of IFN-γ for 24 hours prior to infection with VACV-Luc-GFP. All cells were infected at MOI = 0.01. (E) HSV-1 viral infections. Qualitative images of plaque assay for cells infected with HSV-1 (MOI = 1) with various dilutions. Glc: glucose (25 mM). Gal: galactose (10 mM). Statistical analysis was performed using an unpaired t-test in GraphPad Prism 9.5.1: n.s. not significant, * P ≤ 0.05, ** P< 0.01, *** P ≤ 0.001, **** P ≤ 0.0001. (TIF) [file ppat.1012673.s004.tif]

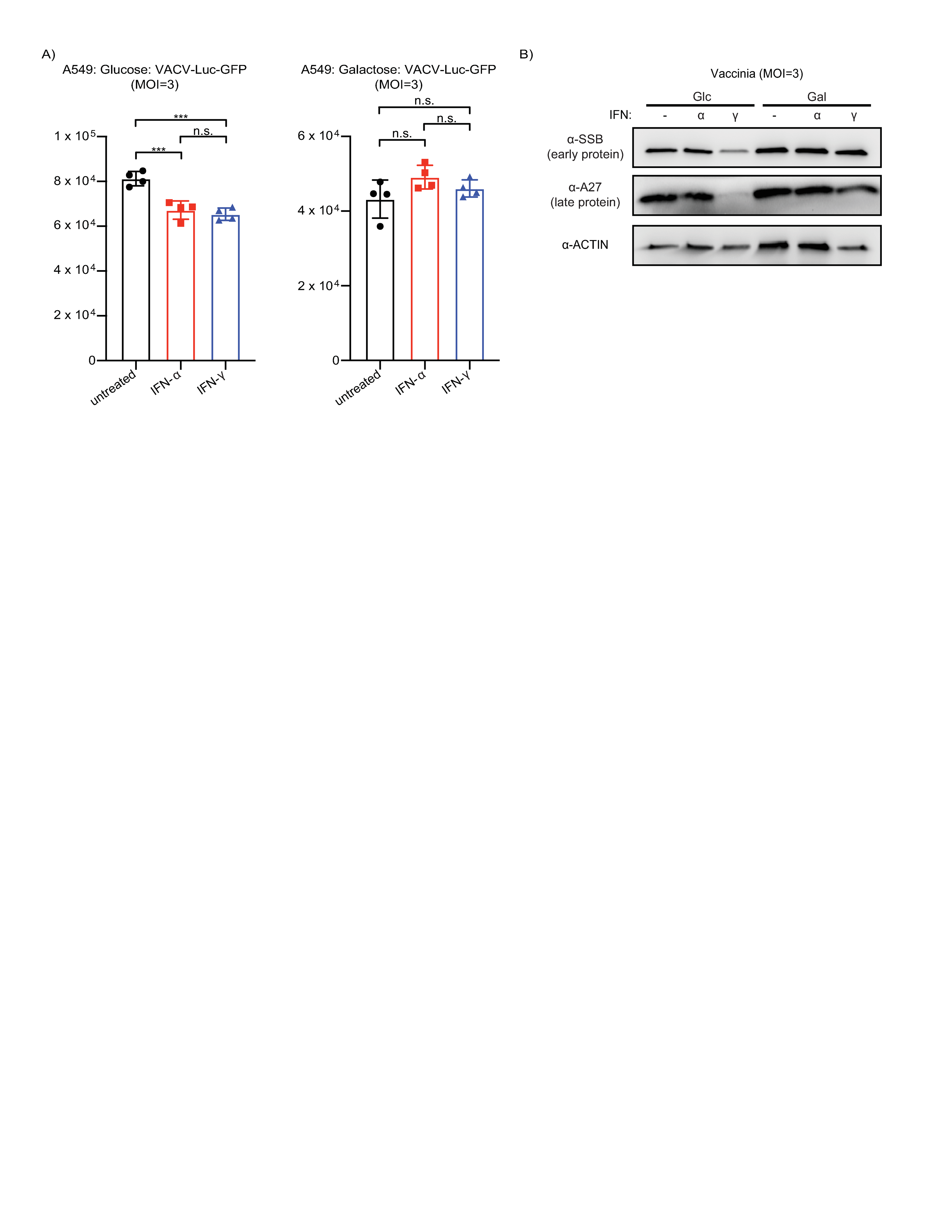

Supplement: S5 Fig — A549 cells were infected with vaccinia virus using the same conditions as in Fig 5 and outlined in S4A Fig but with MOI = 3. (A) Vaccinia virus luciferase reporter assays 24 hours post-infection (N = 4). (B) Western blot of vaccinia virus proteins (SSB: vaccinia virus early protein, A27: vaccinia virus late protein). Lysates were harvested at 24 hours post-infection. Glc: glucose (25 mM). Gal: galactose (10 mM). Statistical analysis was performed using an unpaired t-test in GraphPad Prism 9.5.1: n.s. not significant, * P ≤ 0.05, ** P< 0.01, *** P ≤ 0.001, **** P ≤ 0.0001. (TIF) [file ppat.1012673.s005.tif]

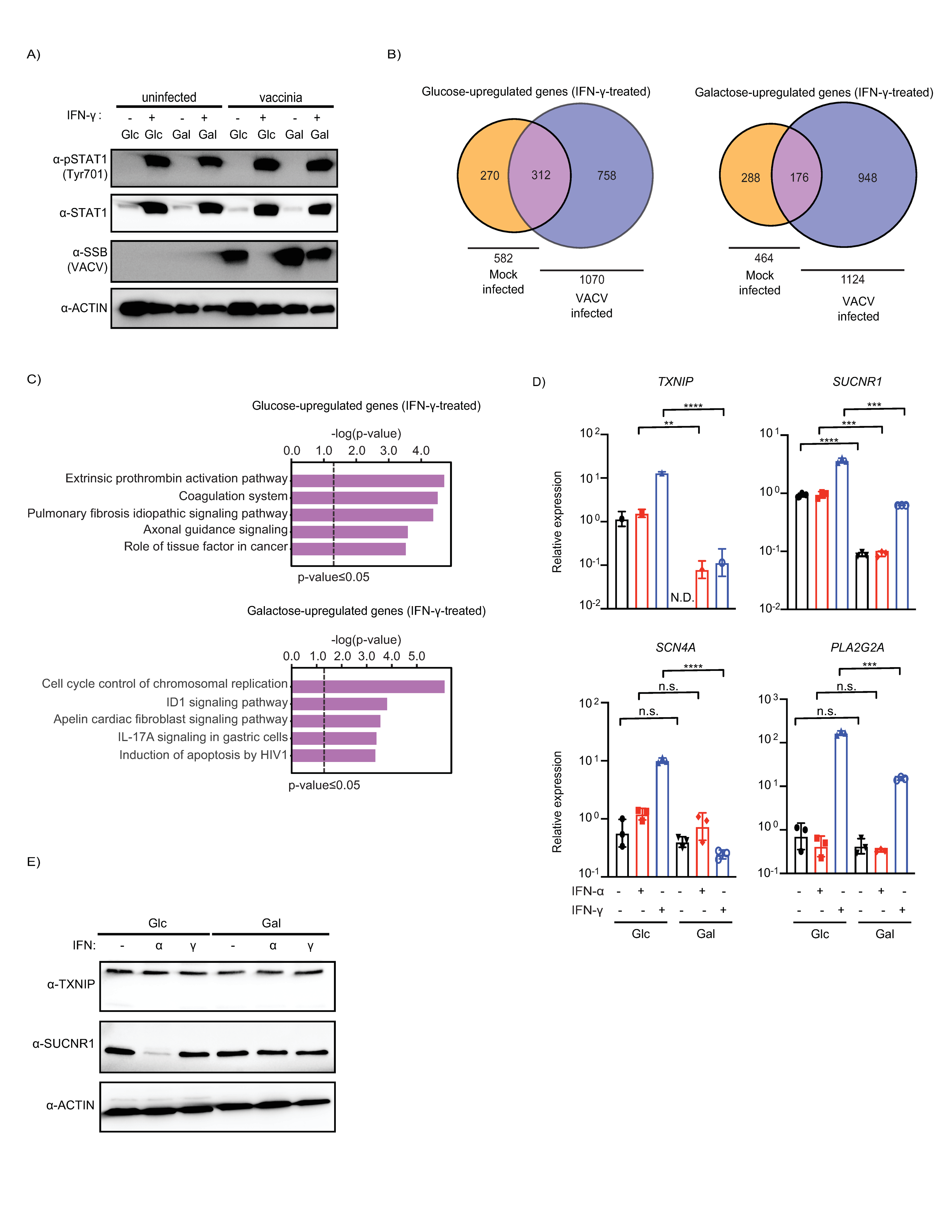

Supplement: S6 Fig — (A) Western blot analysis of total STAT1 and phospho-STAT1 (Tyr 701) in glucose and galactose grown A549 cells, with and without IFN-γ priming, in mock or VACV infected cells. (B) Venn diagram showing the number of differentially expressed genes ‐ log2 fold change ≥ 1 or ≤ -1 and adjusted p-value ≤ 0.01–24 hrs post mock (orange) and VACV infection (blue) (MOI = 0.01) in cells primed with glucose or galactose-media and pre-treated with IFN-γ prior to infection. Left: genes upregulated in glucose relative to galactose; right: genes upregulated in galactose relative to glucose. (C) IPA core canonical pathway analysis for differentially regulated genes (log2 fold change ≥ 1 or ≤ -1 and adjusted p-value ≤ 0.01) shared between mock and VACV infected cells primed with IFN-γ and grown in different carbon sources. Dashed line indicates p-value significant threshold of 0.05. (D) qPCR validation of top gene candidates ‐ from RNA-seq ‐ upregulated in both glucose mock/IFN-γ primed and vaccinia virus/IFN-γ primed infected cells relative to matching conditions but supplemented with galactose; (N = 3). (E) Western blot analysis of top candidate genes. Experiments were carried out according to the timeline outlined in S4A Fig. Statistical analysis was performed using an unpaired t-test in GraphPad Prism 9.5.1: n.s. not significant, * P ≤ 0.05, ** P< 0.01, *** P ≤ 0.001, **** P ≤ 0.0001. (TIF) [file ppat.1012673.s006.tif]

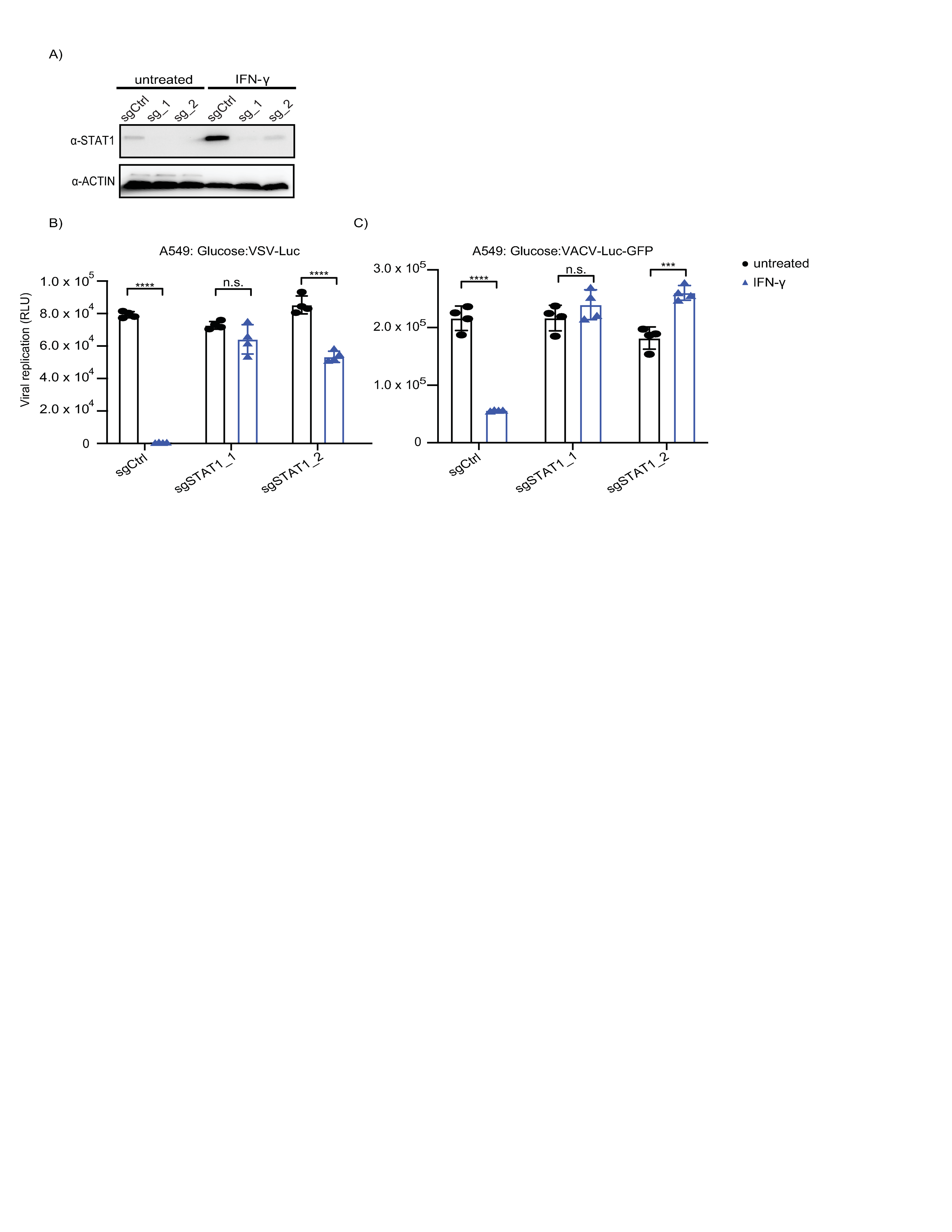

Supplement: S7 Fig — (A) Western blot validation of STAT1 KO A549 cell lines generated with pLentiCRISPR V2 vectors. Two sgRNAs were used per target gene; sgCtrl–non targeting control. (B) Glucose: VSV-Luc (luciferase) replication assays in STAT1 KO cells primed with IFN-γ (N = 4). (C) Glucose: VACV-Luc-GFP (luciferase) replication assays in STAT1 KO cells primed with IFN-γ (N = 4). Experiments were carried out according to the timeline outlined in S4A Fig. Statistical analysis was performed using an unpaired t-test in GraphPad Prism 9.5.1: n.s. not significant, * P ≤ 0.05, ** P< 0.01, *** P ≤ 0.001, **** P ≤ 0.0001. (TIF) [file ppat.1012673.s007.tif]

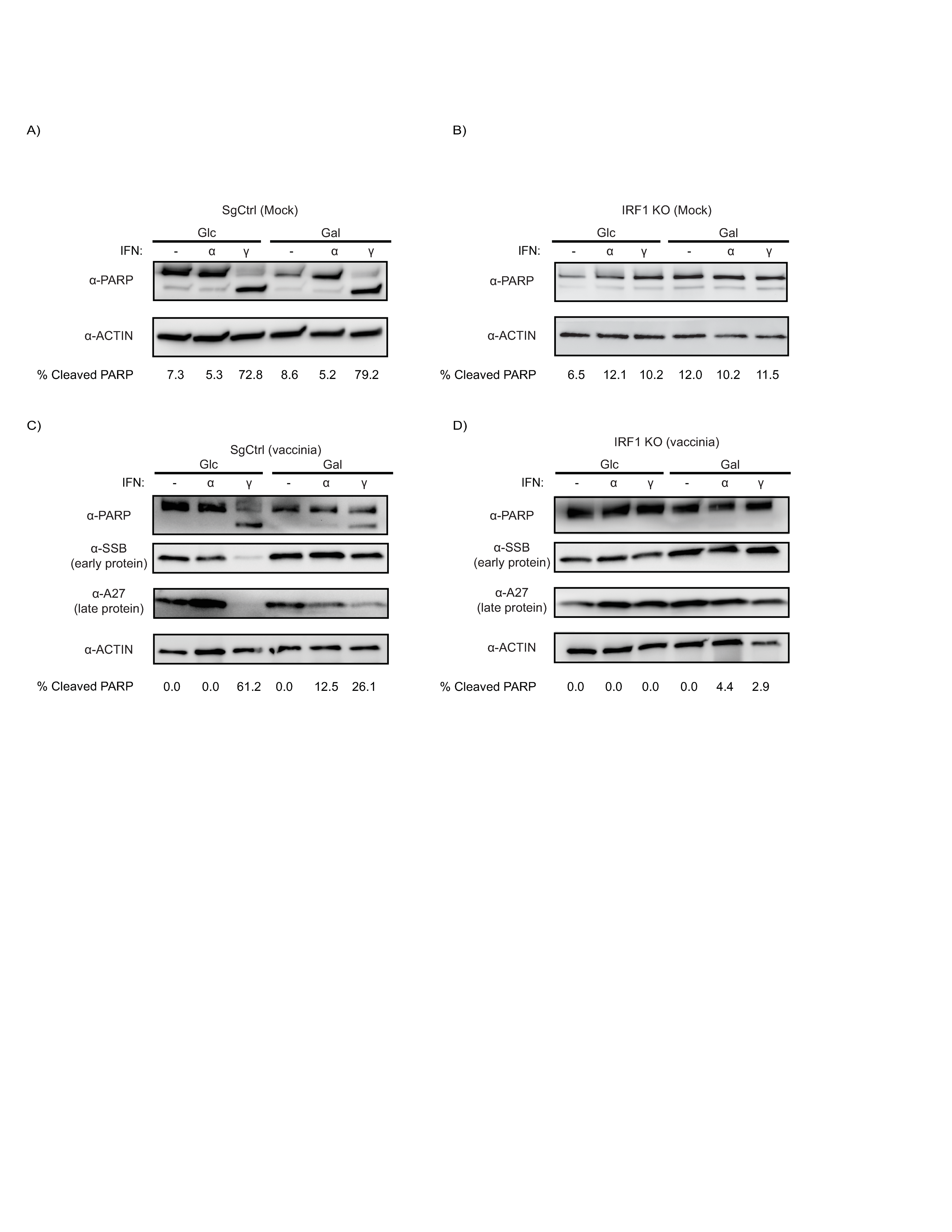

Supplement: S8 Fig — A549 sgCtrl and IRF1 KO cells (sgIRF1_1) were infected with vaccinia virus (MOI = 0.01) with or without interferon-priming. Protein lysates were harvested 24 hours post-infection and analyzed by western blot. PARP western blot analysis from (A) mock infected sgCtrl cells, (B) mock infected IRF1 KO cells, (C) vaccinia infected sgCtrl cells, (D) vaccinia infected IRF1 KO cells. Experiments were carried out according to the timeline outlined in S4A Fig. VACV: vaccinia virus. (TIF) [file ppat.1012673.s008.tif]

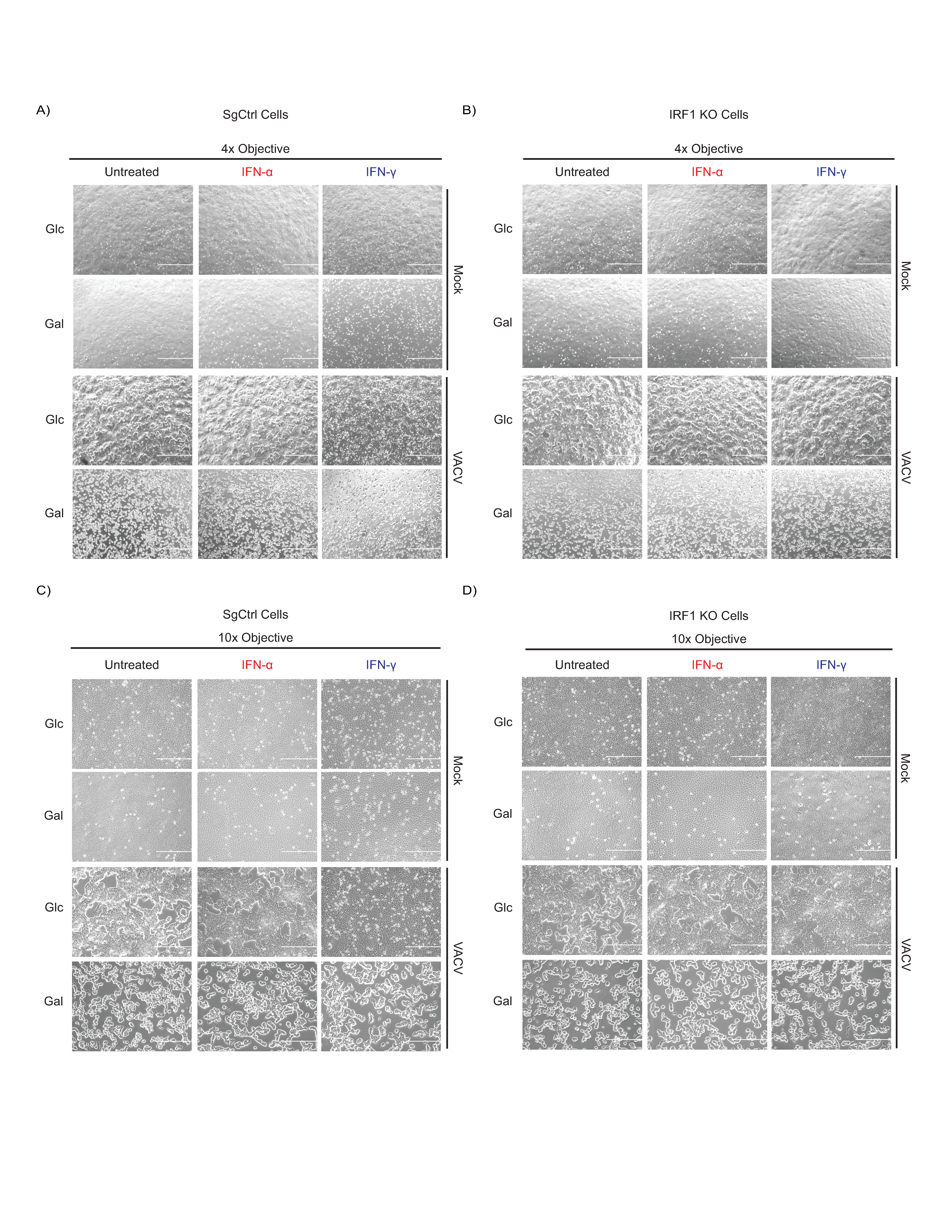

Supplement: S9 Fig — A549 sgCtrl and IRF1 KO (sgIRF1_1) cells ‐ cultured in either glucose/galactose media ‐ were infected with vaccinia virus (MOI = 0.01) with or without interferon-priming. Microscope images were taken 24 hours post-infection. (A) Brightfield microscope images at 4X of both mock and vaccinia virus infected sgCtrl cells primed with glucose/galactose and grown with or without IFNs. (B) Brightfield microscope images at 4X of both mock and vaccinia virus infected IRF1 KO cells primed with glucose/galactose and grown with or without IFNs. (C) Brightfield microscope images at 10X of both mock and vaccinia virus infected sgCtrl cells primed with glucose/galactose and grown with or without IFNs. (D) Brightfield microscope images at 10X of both mock and vaccinia virus infected IRF1 KO cells primed with glucose/galactose and grown with or without IFNs. Experiments were carried out according to the timeline outlined in S4A Fig. VACV: vaccinia virus. (TIF) [file ppat.1012673.s009.tif]

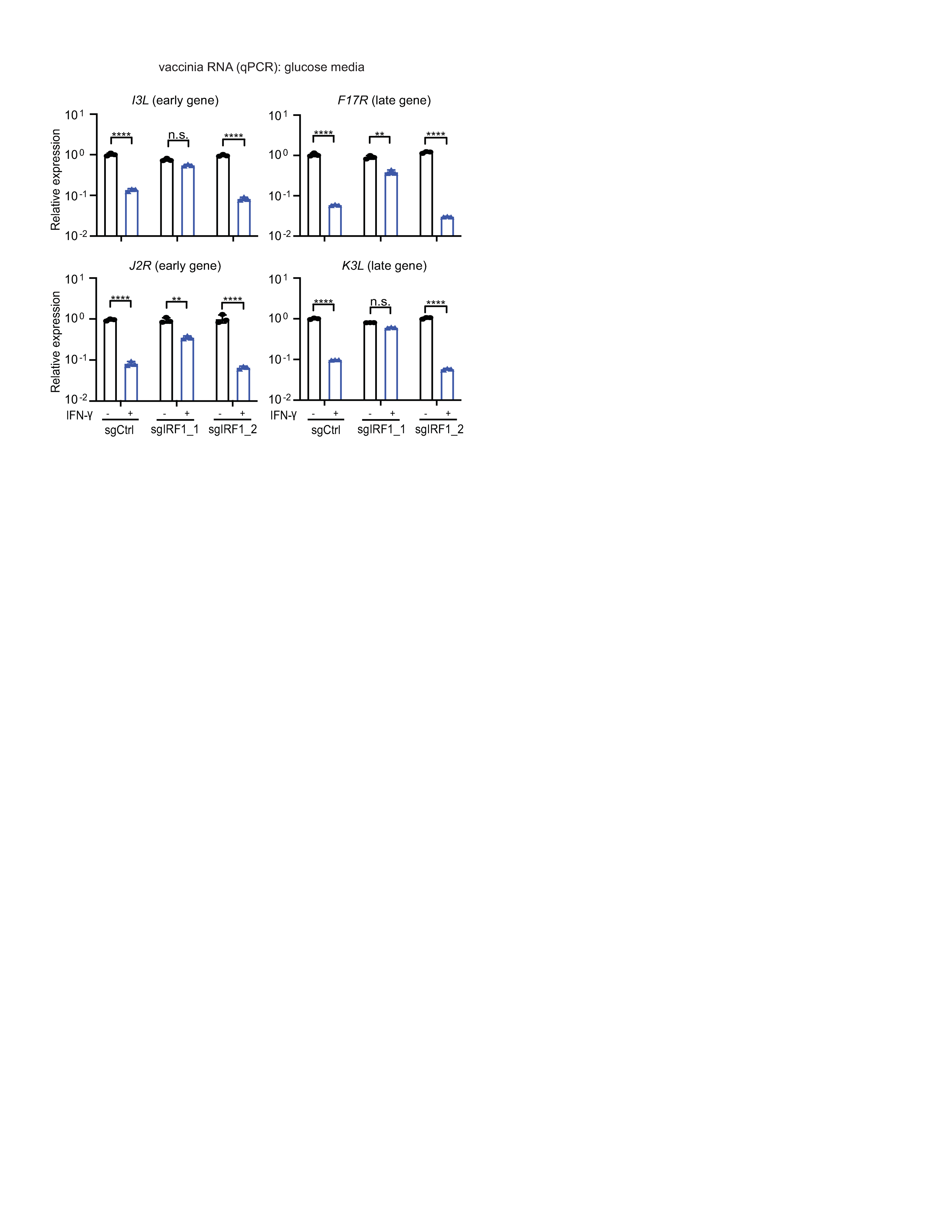

Supplement: S10 Fig — qPCR of VACV transcripts–early (I3L, J2R) and late (F17R, K3L) genes in sgCtrl, IRF1 KO (sgIRF1_1) cells, and sgIRF1_2 where IRF1 KO what is inefficient in glucose/IFN-γ (N = 3). Statistical analysis was performed using an unpaired t-test in GraphPad Prism 9.5.1: n.s. not significant, * P ≤ 0.05, ** P< 0.01, *** P ≤ 0.001, **** P ≤ 0.0001. (TIF) [file ppat.1012673.s010.tif]

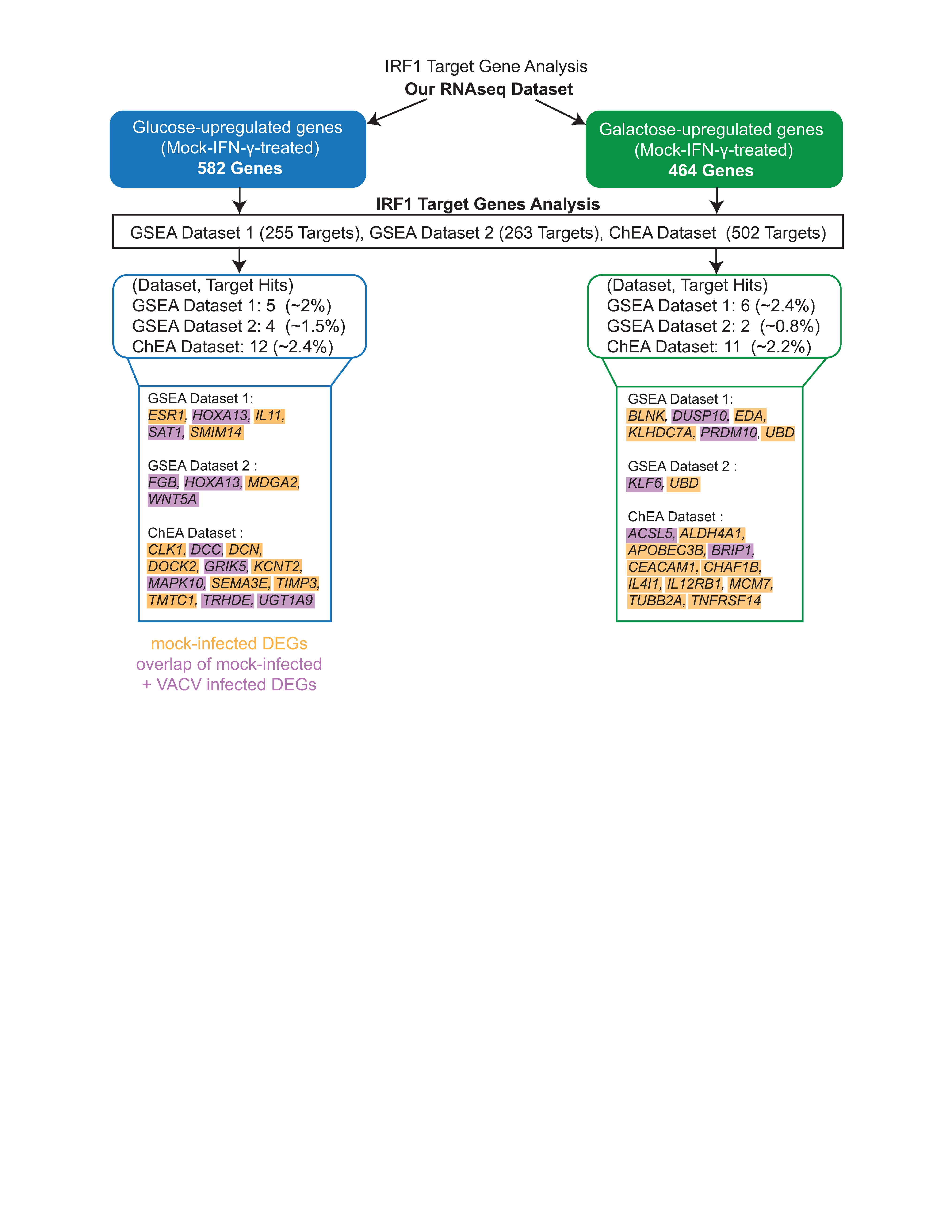

Supplement: S11 Fig — To explore whether there was an IRF1 target gene signature for glucose/IFN-γ cells relative to galactose/IFN-γ cells, we examined differentially expressed genes between the two conditions for evidence of IRF1 regulation. Analysis of three available IRF1 target gene sets, which consist of predicted targets and targets identified from -omics studies (see methods), identified only a limited number of “IRF1 target genes” that differed between our RNA-seq of glucose/IFN-γ cells and galactose/IFN-γ cells. Integrated IRF1 target gene analysis of RNA-seq dataset of glucose/IFN-γ and galactose/IFN-γ cells is shown. Genes highlighted in purple are present under both mock and VACV infected conditions. Genes highlighted in orange are present under only mock-infected conditions. Glucose-upregulated genes in IFN-γ are colored in blue. Galactose-upregulated genes in IFN-γ are colored in blue. See S4 Table. (TIF) [file ppat.1012673.s011.tif]
